# Supplementary material for: Improving 3D convolutional neural network comprehensibility via interactive visualization of relevance maps: evaluation in Alzheimer’s disease
Source: Alzheimers Res Ther. 2021 Nov 23;13:191. doi: 10.1186/s13195-021-00924-2 (PMC8611898; doi:10.1186/s13195-021-00924-2)
Supplement: Supplementary file 2 — Additional file 2: Supplementary Table 2. Group separation performance for hippocampus volume and the convolutional neural network models for raw input data. [file 13195_2021_924_MOESM2_ESM.docx]

Supplementary Table 2 Group separation performance for hippocampus volume and the convolutional neural network models for raw input data.

| **Sample** | **3D convolutional neural network** | |
| --- | --- | --- |
|  | **Balanced accuracy  (mean** ± **SD)** | **AUC  (mean** ± **SD)** |
| **ADNI-GO/2** |  |  |
| MCI vs. CN | *(71.1 % ± 5.7 %)* | *(0.731 ± 0.070)* |
| AD vs. CN | *(84.6 % ± 6.5 %)* | *(0.921 ± 0.024)* |
| MCI^+^ vs. CN^-^ | *(80.7 % ± 7.9 %)* | *(0.881 ± 0.069)* |
| AD^+^ vs. CN^-^ | *(92.4 % ± 3.9 %)* | *(0.974 ± 0.015)* |
| **ADNI-3** |  |  |
| MCI vs. CN | 62.6 % (60.7 % ± 2.6 %) | 0.629 (0.626 ± 0.017) |
| AD vs. CN | 86.1 % (82.1 % ± 5.8 %) | 0.919 (0.907 ± 0.028) |
| MCI^+^ vs. CN^-^ | 71.8 % (70.6 % ± 4.9 %) | 0.769 (0.745 ± 0.021) |
| AD^+^ vs. CN^-^ | 82.2 % (78.8 % ± 5.2 %) | 0.873 (0.877 ± 0.026) |
| **AIBL** |  |  |
| MCI vs. CN | 69.1 % (64.8 % ± 3.2 %) | 0.735 (0.713 ± 0.016) |
| AD vs. CN | 83.7 % (80.2 % ± 6.3 %) | 0.922 (0.924 ± 0.006) |
| MCI^+^ vs. CN^-^ | 78.0 % (73.3 % ± 4.5 %) | 0.837 (0.817 ± 0.025) |
| AD^+^ vs. CN^-^ | 86.3 % (83.7 % ± 6.8 %) | 0.959 (0.959 ± 0.007) |
| **DELCODE** |  |  |
| MCI vs. CN | 69.8 % (69.0 % ± 2.4 %) | 0.779 (0.761 ± 0.017) |
| AD vs. CN | 89.8 % (83.5 % ± 6.0 %) | 0.947 (0.937 ± 0.023) |
| MCI^+^ vs. CN^-^ | 72.5 % (72.5 % ± 5.9 %) | 0.853 (0.814 ± 0.049) |
| AD^+^ vs. CN^-^ | 92.5 % (86.0 % ± 7.1 %) | 0.982 (0.967 ± 0.028) |

Reported values are the respective measures for the single model trained on the whole ADNI-GO/2 dataset. In parenthesis, the mean values and standard deviation for the ten models trained in the tenfold cross-validation procedure are provided to indicate the variability of the measures. Values for the ADNI-GO/2 sample (in italics) may be biased as the respective test subsamples were used to determine the optimal model during training. We still report them for better comparison of the model performance across samples.
