# Supplementary figures and images for: Improving 3D convolutional neural network comprehensibility via interactive visualization of relevance maps: evaluation in Alzheimer’s disease
Source: Alzheimers Res Ther. 2021 Nov 23;13:191. doi: 10.1186/s13195-021-00924-2 (PMC8611898; doi:10.1186/s13195-021-00924-2)

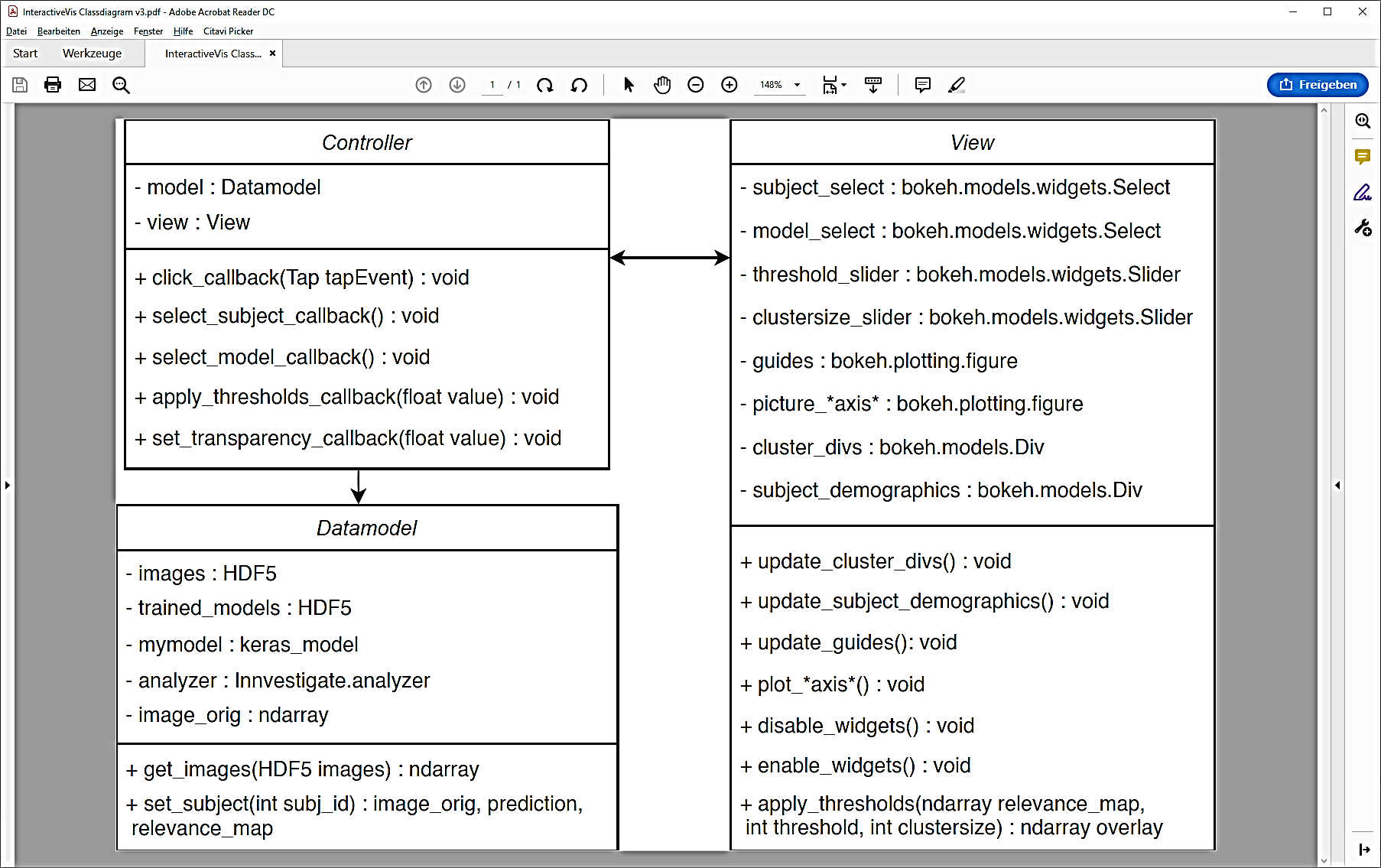


Supplementary Figure 1 UML diagram of the interactive visualization application.

Supplement: Supplementary file 3 — Additional file 3: Supplementary Figure 1. UML diagram of the interactive visualization application. [file 13195_2021_924_MOESM3_ESM.docx]

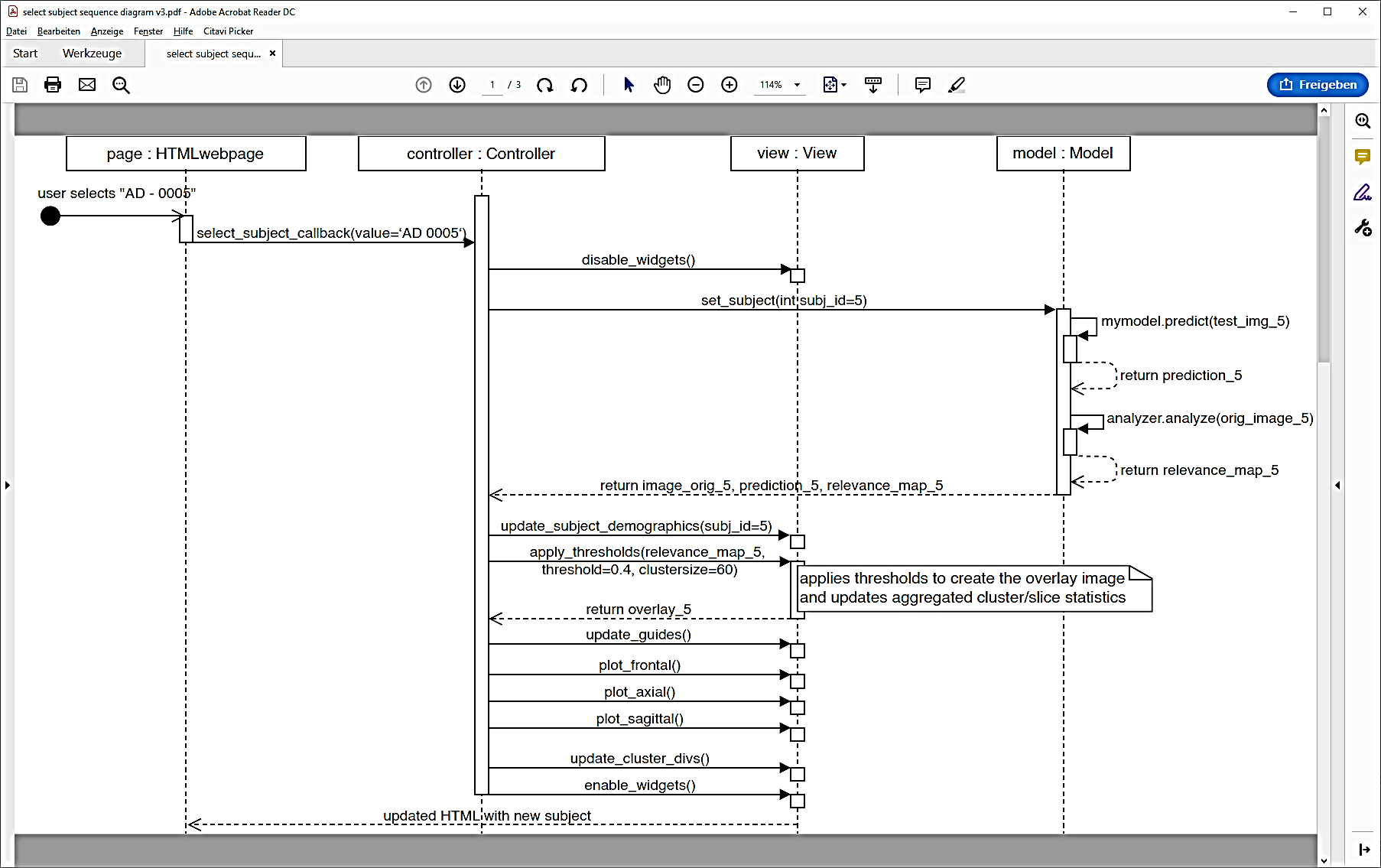


Supplementary Figure 2 Sequence diagram of function calls when selecting a new person.

Supplement: Supplementary file 4 — Additional file 4: Supplementary Figure 2. Sequence diagram of function calls when selecting a new person. [file 13195_2021_924_MOESM4_ESM.docx]
