# Supplementary material for: Improving 3D convolutional neural network comprehensibility via interactive visualization of relevance maps: evaluation in Alzheimer’s disease
Source: Alzheimers Res Ther. 2021 Nov 23;13:191. doi: 10.1186/s13195-021-00924-2 (PMC8611898; doi:10.1186/s13195-021-00924-2)
Supplement: Supplementary file 5 — Additional file 5: Supplementary Figure 3. Comparison of mean relevance maps between samples. [file 13195_2021_924_MOESM5_ESM.docx]

DELCODE

ADNI-GO/2


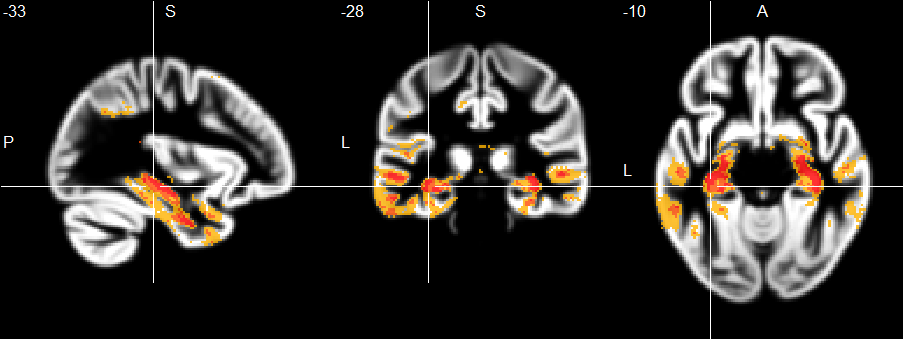

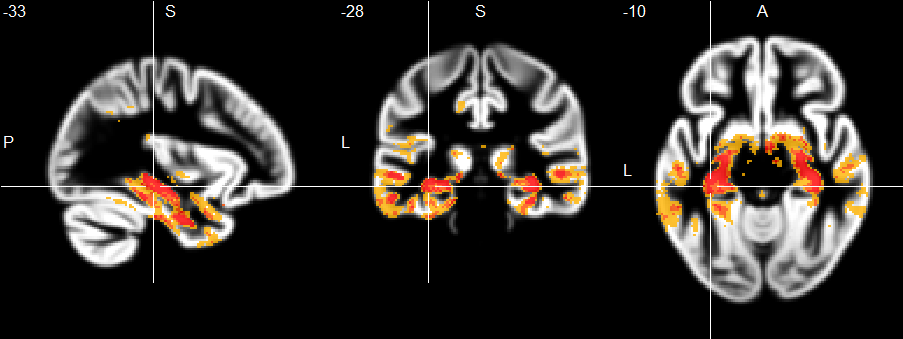

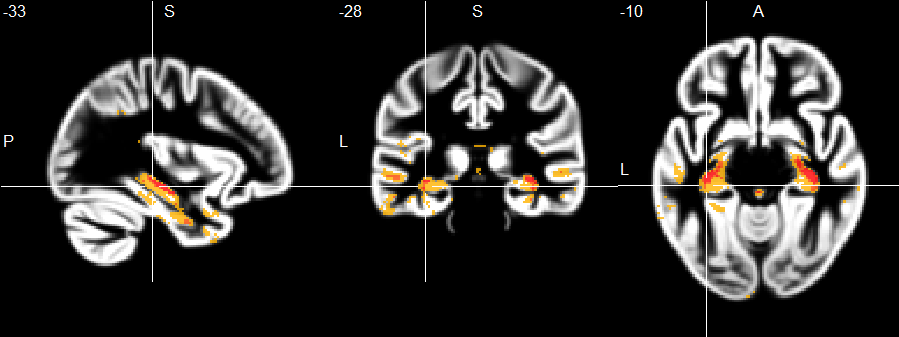

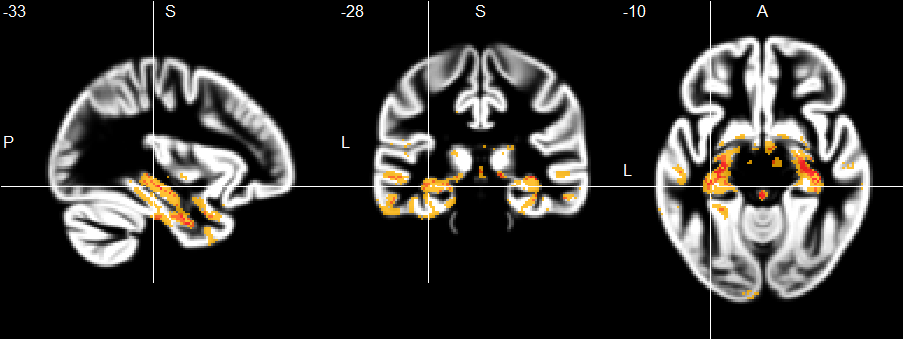


Alzheimer’s dementia

Mild cognitive impairment


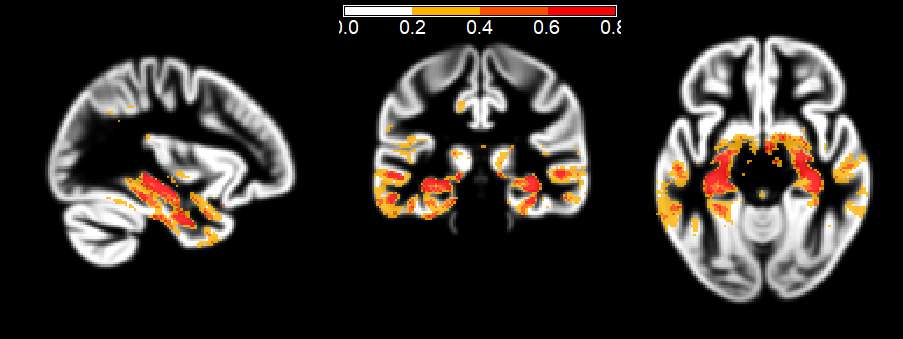

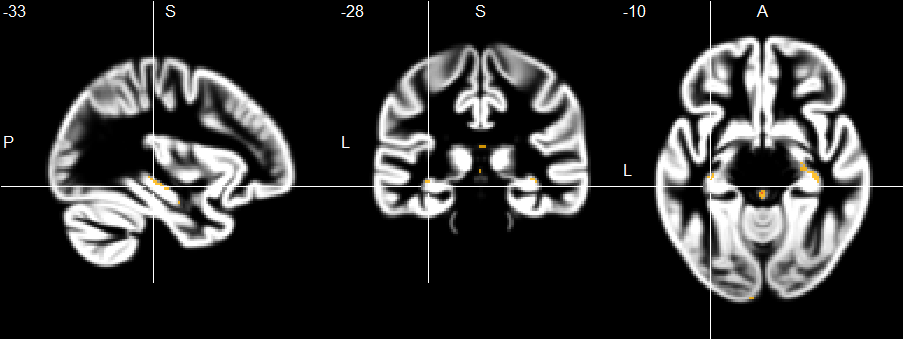

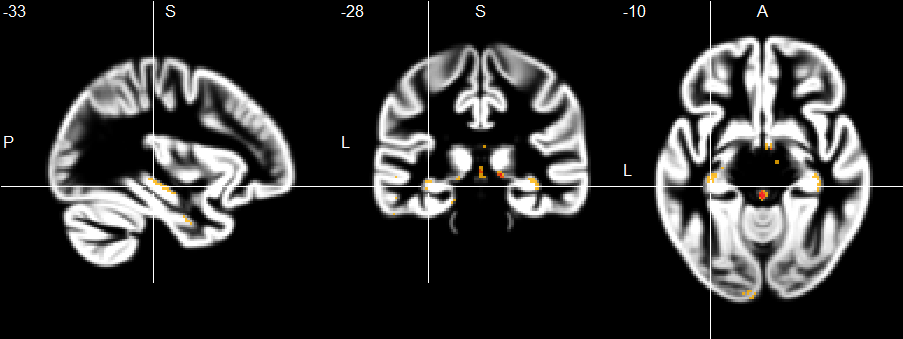


Healthy control

Supplementary Figure 3 Comparison of mean relevance maps between samples. Left: ADNI-GO/2, Right: DELCODE.
