# Supplementary material for: Improving 3D convolutional neural network comprehensibility via interactive visualization of relevance maps: evaluation in Alzheimer’s disease
Source: Alzheimers Res Ther. 2021 Nov 23;13:191. doi: 10.1186/s13195-021-00924-2 (PMC8611898; doi:10.1186/s13195-021-00924-2)
Supplement: Supplementary file 6 — Additional file 6: Supplementary Figure 4. Correlation matrix of hippocampus volume (residualized) and several brain regions’ relevance scores for DELCODE participants and the model trained on the whole ADNI-GO/2 dataset. [file 13195_2021_924_MOESM6_ESM.docx]

**
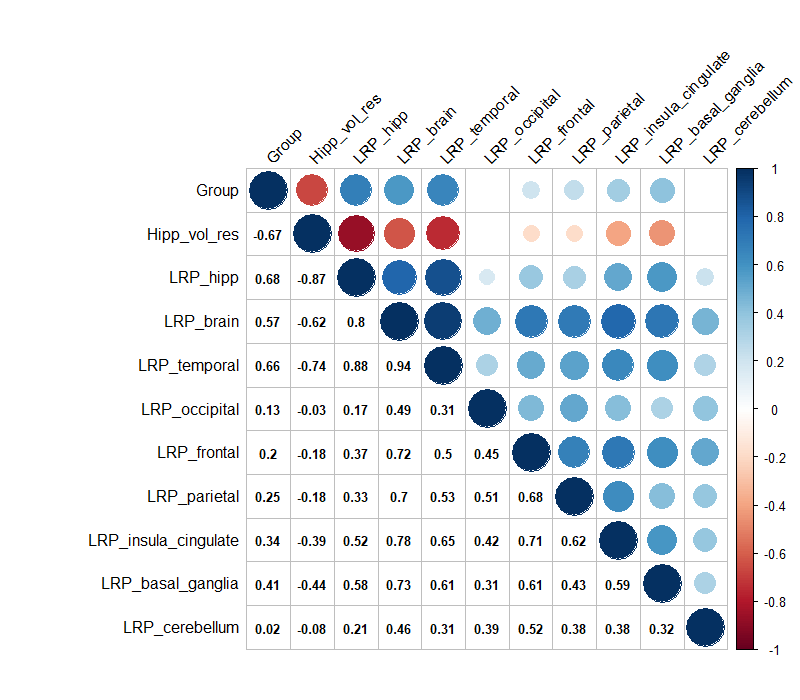
**

Supplementary Figure 4 Correlation matrix of hippocampus volume (residualized) and several brain regions’ relevance scores for DELCODE participants and the model trained on the whole ADNI-GO/2 dataset. The correlation between hippocampus volume and hippocampus relevance was highest (-0.87). Upper right triangle entries were thresholded a p<0.001. For simplicity, group was numerically encoded as CN=1, MCI=2, AD=3.
